# Alcohol extracts of Chinese bayberry branch induce S‐phase arrest and apoptosis in HepG2 cells

**DOI:** 10.1002/fsn3.3080

**Published:** 2022-10-02

**Authors:** Yuanyuan Zheng, Zheping Yu, Yougui Li, Shi Zhong, Yuqing Sun, Li Sun, Xiliang Zheng, Xingjiang Qi, Shuwen Zhang

**Affiliations:** ^1^ Institute of Horticulture Zhejiang Academy of Agricultural Sciences Hangzhou China; ^2^ Institute of Sericultural and Tea Zhejiang Academy of Agricultural Sciences Hangzhou China; ^3^ Xianghu Lab Hangzhou China

**Keywords:** apoptosis, Chinese bayberry, HepG2 cells, MRBE, S‐phase arrest

## Abstract

The alcohol extracts of Chinese bayberry (*Myrica rubra*) branches (MRBE) are rich in flavonoids which have a variety of medicinal benefits, but their effects on human HepG2 were unknown. In this study, the effects of MRBE on HepG2 cell growth and its potential for inhibiting cancer were explored. The results displayed that MRBE inhibited HepG2 proliferation both by arresting cells in S phase and promoting apoptosis. Quantitative reverse‐transcription PCR (qRT‐PCR), western blotting, and immunofluorescence showed that MRBE induced S‐phase arrest by upregulating *p21*, which in turn downregulated cyclin and cyclin‐dependent kinase messenger RNA (mRNA) and protein. Apoptosis was induced by blocking the expression of *BCL‐2* and suppression of the *Raf*/*ERK1* signaling pathways. These results indicated that MRBE may have the potential for treatment of human liver cancer, highlighting novel approaches for developing new pharmacological tools for the treatment of this deadly type cancer. Meanwhile, it provides a new direction for the medicinal added values of Chinese bayberry, which helped to broaden the diversified development of its industry chain.

## Graphical abstract

The alcohol extracts of Chinese bayberry *Myrica rubra* branches (MRBE) effectively inhibited the proliferation of HepG2 cells by inducing cell cycle arrest in S phase and promoting apoptosis, with this arrest potentially mediated by the activation of p21–Cyclin–CDK (cyclin‐dependent kinase) complex signaling pathways. This study indicated that MRBE may have the potential for the treatment of human liver cancer.

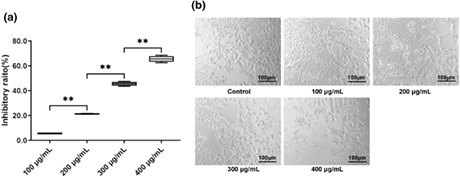

## 1 INTRODUCTION

Hepatocellular carcinoma (HCC) is the most common variety of primary liver cancer (Kim et al., 2017). The 2021 cancer statistics showed that although liver cancer prevalence appears stable in males, it has risen in females, primarily due to obesity, hepatitis B and C viral infection, and other factors (Siegel et al., 2021). Chemotherapy, surgical resection, and other treatment methods can improve patient survival, but up to 70% of these patients nonetheless experience recurrent disease within five years (Chen et al., 2016). Additionally, most patients are diagnosed when the cancer progressed and survived just 6–8 months on average (Gosalia et al., 2017). Owing to its initially indolent course, delayed symptom onset, and limited treatment options (Zhu et al., 2016), HCC remains a considerable threat, and its prevention and treatment are areas of active research interest.

Chinese bayberry (*Myrica rubra* Sieb. et Zucc.) is a famous subtropical fruit tree, which is mainly grown in Southern China, such as Zhejiang, Fujian, Yunnan provinces, etc., and it has important economic values. The cultivation of Myrica plants has been for more than 2000 years according to the historical records in China. The bayberry fruit has a unique sweet/sour flavor and is beneficial to health, as it contains high levels of phenolic compounds, ascorbic acid, and anthocyanins (Sun et al., 2013). In addition, different organs of Chinese bayberry have high medicinal values. Previous studies have showed that active substance from bayberry fruit can protect against oxidative DNA damage (Chen, Liu, et al., 2015), exhibit robust antioxidant activity (Huang et al., 2014), and prevent hypoglycemia (Zhang et al., 2015). Bayberry leaf extracts offer protection against virus and bacterial infections as well as oxidative stress (Chen, Liu, et al., 2015; Zhang et al., 2015), and exhibit bacteriostatic (Zhang et al., 2017), antioxidant and antiproliferative (Zhang et al., 2016), neuroprotective (Li et al., 2018), and hypoglycemic activities (Wang, Jiang, et al., 2019; Zheng et al., 2021). In the cultivation of Chinese bayberry, the tree needs to be trimmed 2–3 times/year, and the trimmed branches become waste. Old trees need to be cut down, and are also discarded or burned, resulting in both waste and pollution. With the continuous improvement of cultivation efficiency and the continuous expansion of the planting area, the potential resources of branches are also accumulating and getting wasted. The functions of ethanol extracts prepared using bayberry branches, however, have not been reported to date.

Most anticancer drugs were originally from natural plants (Li & Martin, 2011; Rawat et al., 2018). Traditional Chinese medicine has utilized natural products to treat diseases for thousands of years, with most plant parts being used as sources of these natural compounds, which largely derive their pharmacological activity from plant secondary metabolites (Beyoğlu & Idle, 2020; Yu et al., 2021). Many drugs and natural products used in the treatment of liver cancer modulate enzyme levels or cell signaling pathways. For example, Tatariside F (TF), which is extracted from the roots of *Fagopyrum tataricum* (L.)Gaertn, exhibits significant antitumor activity against HCC through a mechanism that may be related to increased expression of *p53* and *BAX* and reductions in Bcl‐2 (B‐cell lymphoma 2) protein levels (Peng et al., 2015). Piperlongumine (PL) is a natural extract of piperlongumine that selectively kills HCC cells and preferentially inhibits both invasion and migration of HCC cells via the ROS–ER–MAPK–CHOP (reactive oxygen species–endoplasmic reticulum–mitogen‐activated protein kinase–c/EBP homology protein) signaling pathway (Chen, Zhou, & Zheng, 2015). The ethanol extract of *Artemisia capillaris* leaves has been shown to not only effectively promote apoptosis, but to also reduce human HCC cell growth by suppressing PI3K/AKT (phosphoinositide 3‐kinase/protein kinase B) signaling activity (Kim et al., 2018). Other studies have explored the effects of proanthocyanidins on proteins associated with apoptosis, the cell cycle, and MAPK signaling and on the expression of NAG‐1 (nonsteroidal anti‐inflammatory drug‐activated gene‐1) in HepG2 cells (Wang et al., 2020). In addition, natural extracts from plants, such as *Beilschmiedia tsangii* root (Chen et al., 2021), *Kigelia africana* (Lam.) Benth. (Wambua Mukavi et al., 2020), *Diospyros kaki* leaves (Ko et al., 2020), Licorice (Wang, Luo, et al., 2019), *Phoenix dactylifera* L. (Khan et al., 2017), germ and bran of red rice (Upanan et al., 2019), *Coptis chinensis* (Kim et al., 2021), and *Juniperus communis* Linn. (Huang et al., 2021), have been found to induce the apoptotic death of HepG2 cells via the exogenous, mitochondria‐mediated endogenous pathway, MAPK signaling pathway, and ER signaling pathway. Thus, these extracts have the potential for being developed into drugs that specifically target HCC.

In this study, MRBE was extracted from *Myrica rubra* branches via an ultrasonic ethanol extraction method. To explore the molecular mechanisms whereby it induces apoptosis, the application of different concentrations of the extracts to HepG2 cells and related experiments were carried out.

## 2 MATERIALS AND METHODS

### 2.1 MRBE preparation and targeted metabolite analysis

MRBE was extracted from Chinese bayberry waste branches collected after pruning. The trees were planted in the International Research Center of Chinese bayberry (latitude 29.30°N, longitude 119.59°E), Jinhua, Zhejiang Province, China. This center was cofounded by our institute and local government, and the age of most trees used in this study was over 15 years.

The fresh branches of “Dongkui” bayberry were selected and removed from leaves, and dried to a constant weight at 60°C. Samples were crushed and ground, and then filtered using 70% ethanol for ultrasonic extraction at room temperature 4 times (filter pore size 80–120 μm). After repeated evaporation and freeze‐drying, MRBE was obtained from these extracts for subsequent study. All experiments were performed using three biological duplicate samples.

Next, 50 mg of the MRBE sample was added to 600 μl of water:methanol (v:v = 1:2) containing succinic acid‐2,2,3,3‐d4 (50 ng/ml), after which 400 μl of chloroform was added. Two steel balls were then added, and the samples were pulverized in a grinder at 60 Hz for 2 min (60 Hz). The material was then sonicated on ice for 20 min. Samples were centrifuged for 10 min (4°C, 13,000 *g*), and 500 μl of the supernatant was placed in an Eppendorf (EP) tube. The residue was added to 400 μl of water: methanol (v:v = 1:2) containing succinic acid‐2,2,3,3‐d4 (50 ng/ml), after which samples were vortexed, resonicated, and centrifuged (10 min, 4°C, 13,000 *g*), and 300 μl of the resultant supernatant was combined with that prepared above for a total volume of 800 μl, of which 200 μl of supernatant was evaporated, dried, and suspended in 200 μl of water:methanol (v:v = 18:7) including L‐2‐chlorophenylalanine (10 ng/ml) as an internal standard. Samples were then vortexed for 30 s, ultrasonicated for 2 min, and allowed to stand at −20°C for 2 h. After centrifugation for 5 min (4°C, 13,000 *g*), 200 μl of the supernatant was placed in a brown levocarnitine (LC) injection vial through an organic phase pinhole filter (0.22 μm) and stored (−80°C). A UPLC–ESI–MS/MS (ultra‐performance liquid chromatography–electrospray ionization–tandem mass spectrometry) analysis was then performed.

The peak area of each chromatographic peak corresponded to the relative content of the corresponding metabolite, with the integral peak area for each metabolite being used to calculate the concentration thereof based on a standard curve. The absolute content of each metabolite in the actual sample was then determined as follows: Metabolite content (ng/g) = C × V/M × N, where C = the metabolite concentration as calculated with a standard curve based on the peak area value (ng/ml), V: constant volume (0.2 ml), M: sample mass (g), and N: dilution multiplier (5 times).

### 2.2 Cells and kits

The human HepG2 cell line was obtained from the Institute of Biochemistry and Cell Biology of the Chinese Academy of Sciences and grown in Roswell Park Memorial Institute Medium (RPMI) 1640 with 10% fetal bovine serum (FBS), penicillin (100 μg/ml), and streptomycin (100 μg/ml; all Thermo Fisher) at 37°C in a 5% CO_2_ incubator. MTT (3‐[4,5‐Dimethylthiazol‐2‐yl]‐2,5‐diphenyltetrazolium bromide) was purchased from Sigma‐Aldrich and an fluorescein iosthiocyanate (FITC)–Annexin V and propidium iodide (PI) apoptosis kit was from Thermo Fisher.

### 2.3 Cell proliferation assay

Logarithmic‐phase cells were cultured in 96‐well plates for 24 h, after which 100 μl of MRBE stock solution was added to obtain final concentrations of 100, 200, 300, and 400 μg/ml. The control was 0.1% dimethyl sulfoxide (DMSO) in water. The cells were grown for 24, 36, and 48 h, the medium was removed, and the cells were trypsinized. Approximately 20 μl of cell suspensions was mixed with an equal volume of trypan blue solution (0.4%) and counted (IC1000; Countstar; ALIT Life Science Co., Ltd.). Proliferation was measured in an MTT assay and calculated according to the formula “Inhibitory ratio (%) = [1 − absorbance (test)/absorbance (control)] × 100%” (Zhong et al., 2020).

### 2.4 Cell cycle and apoptotic analyses

Cells (1 × 10^5^ cells/well) were grown in the presence of 0, 200, or 400 μg/ml MRBE for 48 h. The cell cycle proportions were examined by flow cytometry (Cytomics FC 500 MCL Flow Cytometer System; Beckman Coulter, Inc.) and analyzed with MultiCycle AV software (CXP V2.3 WIN7, C30309; Phoenix Flow Systems, Inc.). Apoptosis was evaluated by Annexin V–FITC (5 μl) and PI (5 μl) staining at 4°C for 15 min followed by flow cytometry. Data were from three biological replicates (Zhong et al., 2020).

### 2.5 qRT‐PCR

Total RNA was isolated from cells using the TaKaRa MiniBEST Universal RNA Extraction Kit (Takara). A PrimeScript RT reagent kit with gDNA Eraser (Takara) and SYBR® Fast qPCR Mix (Takara) were used for PCR amplification using a CFX96 real‐time PCR instrument (Bio‐Rad) with the following thermocycler settings: 95°C for 30 s; 40 cycles of 95°C for 5 s; and 60°C for 30 s. β‐Actin served as a normalization control, and the comparative 2^−∆∆Cq^ method was used to compare gene expression using primers detailed in a prior study (Yu et al., 2021). All primers used were presented in Table S1.

### 2.6 Western blotting

Total protein was extracted from cells using SD‐001 buffer (Invent Biotechnologies, Inc.) with protease inhibitors (Beijing Solarbio Science & Technology Co., Ltd.), and the protein concentrations were determined by the bicinchoninic acid (BCA) assay. Proteins were separated on 4%–12% sodium dodecyl sulfate‐polyacrylamide gel electrophoresis (SDS‐PAGE) and transferred to polyvinylidene difluoride (PVDF) membranes which were blocked for 1 h with 5% bovine serum albumin (BSA) at room temperature. Blots were then incubated overnight with primary antibodies (1:1000; Abcam) against p21 (ab188224), Cyclin D (ab28283), CDK4 (cyclin‐dependent kinase 4; ab131469), CDK6 (cyclin‐dependent kinase 6; ab131469), Bcl‐2 (ab32124), and β‐actin (ab8226) at 4°C. Blots were washed in phosphate‐buffered saline (PBS) and incubated for 1 h at room temperature with secondary AF790‐linked goat antirabbit immunoglobulin G (IgG; 1:10,000; ab175781). After further washes, a ChemiDoc Touch Imaging System (Bio‐Rad) was used for protein band visualization with the Image Lab Touch software (v1.2; Chameleon Power).

### 2.7 Immunofluorescence

Cells were grown on slides, fixed for 30 min with 4% paraformaldehyde in PBS at room temperature, and washed in PBS. After blocking with 3% BSA for 50 min, the slides were probed overnight with primary antibodies against p21 (ab188224), cyclin D (ab28283), CDK4 (ab131469), CDK6 (ab131469), and Bcl‐2 (ab32124) at 4°C. After washing, the slides were incubated with DyLight 488‐conjugated goat antirabbit immunoglobulin G (IgG; 1:100; ba1127; Wuhan Boster Biological Technology, Ltd.) for 1 h at room temperature and mounted with 4′,6‐diamidino‐2‐phenylindole (DAPI)‐containing medium (C1002; Beyotime Institute of Biotechnology) to counterstain nuclei and observed under a fluorescence microscope (Nikon Eclipse 80i; Nikon Corporation).

### 2.8 Statistical analysis

Data were expressed as means ± SD and analyzed with SPSS 16.0. One‐way analyses of variance (ANOVAs) and Tukey's post hoc test were used to compare groups. A value of *p* < .05 was the significance threshold.

## 3 RESULTS AND DISCUSSION

### 3.1 UPLC–ESI–MS/MS analysis of MRBE composition

Natural products have proved to be extremely valuable for identifying and developing new drugs (Harvey et al., 2015). The extracts of Chinese bayberry fruits and leaves are rich in antioxidants and have roles to protect against oxidative DNA damage, exhibit high antioxidant activity, and prevent hypoglycemia (Zhang et al., 2015, 2017). However, to our knowledge, the effects of MRBE on HCC development have not been demonstrated to date. In this study, MRBE accounted for about 23% of the dry weight of branches (280 g/1200 g) and was found to be primarily composed of 12 main flavonoids and 68 components: anthocyanins (2), benzoic acid derivatives (13), catechin derivatives (8), coumarins (4), dihydrochalcones (2), flavanones (6), flavones (5), flavonols (15), phenylpropanoids (9), proanthocyanidins (1), stilbenes (2), and terpenoids (1) (Table 1). The most abundant component was gallic acid (1,122,478.5 ng/g), followed by myricitrin (659,828.5 ng/g). These components may thus be bioactive compounds that shaped the activity of MRBE.

**TABLE 1:** The results of UPLC–ESI–MS/MS (ultra‐performance liquid chromatography–electrospray ionization–tandem mass spectrometry) analyses of *Myrica rubra* branches' extraction (MRBE)

Component name	Classification	Content (ng/g)	Component name	Classification	Content (ng/g)	Component name	Classification	Content (ng/g)
Gallic acid	Benzoic acid derivatives	1,122,478.5	Myricitrin	Flavonols	659,828.5	4‐Hydroxycinnamic acid	Phenylpropanoids	6769.4
Gentisic acid		189,624.4	Quercitrin		127,855.2	Coniferaldehyde		3039.5
Salicylic acid		160,769.4	Myricetin		59,566.7	Chlorogenic acid		2175
Syringic acid		142,955	Myricetin 3‐galactoside		43,578.4	Caffeic acid		1709.9
Vanillic acid		30,105.6	Quercetin 3‐galactoside		15,630.2	Caftaric acid		700.7
trans‐Cinnamic acid		26,914.4	Afzelin		6636	Ferulic acid		455
Ellagic acid		20,416.5	Aromadendrin		4647.5	Cryptochlorogenic acid		447
Vanillin		17,001.2	Astragalin		2765.9	Sinapaldehyde		277
Syringaldehyde		8290.5	Isorhamnetin		637.2	Sinapic acid		196.4
Methyl gallate		4538.4	Quercetin 3‐O‐glucuronide		553.2	Procyanidin B2	Proanthocyanidins	324.3
4‐Hydroxybenzoic acid		1743.5	Nicotiflorin		395.1	4‐Methylumbelliferone	Coumarins	394.6
Acetovanillone		441.2	Avicularin		329.5	Aesculin		343.2
Salicin		46.3	Isorhamnetin‐3‐O‐glucoside		261.5	Daphnetin		332.2
Gallocatechin	Catechin derivatives	205,632.7	Rutin		254.6	Psoralen		126
Epigallocatechin gallate		186,575.1	Kaempferol		156.5	Phlorizin	Dihydrochalcones	1286.6
(−)‐Epigallocatechin		162,507.2	Morin	Flavones	4458.6	Phloretin		21
(−)‐Gallocatechin gallate		37,778.6	Quercetin		4287.4	Eriodictyol	Flavanones	1430
Protocatechuic acid		3097.8	Luteolin		1204.5	(S)‐Pinocembrin		1149.1
Epicatechin		1830	Chrysin		41.4	Prunin		792.1
3,4‐Dihydroxybenzaldehyde		623.5	6‐Methoxyflavone		35.1	Naringenin		590.6
Catechin		192.1	Resveratrol	Stilbenes	8525.4	Isosakuranetin		141
Delphinidin 3‐glucoside	Anthocyanins	53,855.1	trans‐Piceid		236	Sakuranetin		135.1
Cyanidin 3‐O‐rutinoside chloride		666.9	Perillyl alcohol	Terpenoids	8405.1			

### 3.2 MRBE inhibits HepG2 cell proliferation

**FIGURE 1:**
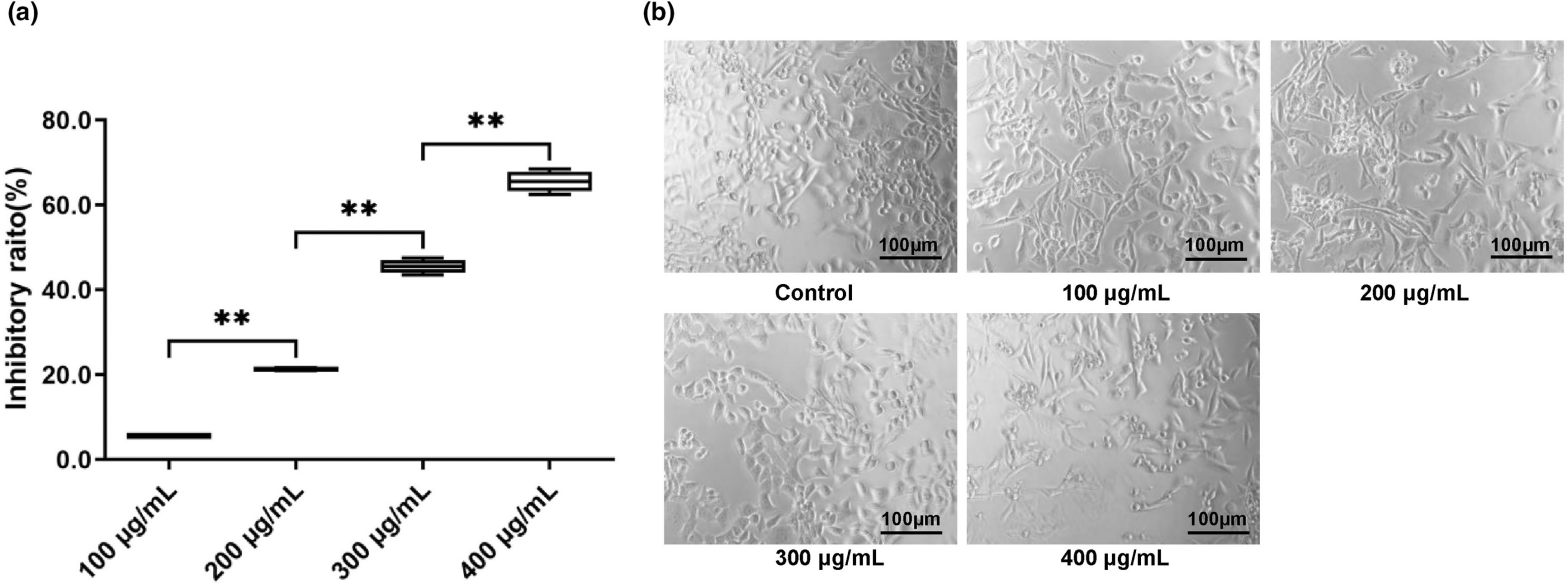
Inhibition of MRBE on HepG2 cell proliferation in vitro. (a) The inhibitory ratio of HepG2 cells treated with MRBE (100, 200, 300, and 400 μg/ml) for 48 h. (b) Morphological observation of HepG2 cells treated with MRBE (100, 200, 300, and 400 μg/ml) for 48 h. Scale bar, 100 μm. Values are presented as the mean ± SD (*n* = 5 each group). **p < .01. MRBE, *Myrica rubra* branches extraction.

Cancers are characterized by continuous cell proliferation, and as such, cell cycle disruption may hinder this process (Moussa et al., 2019). To investigate the effects of MRBE on HepG2 cells, these cells were cultured with varying amounts of MRBE (0, 100, 200, 300, or 400 μg/ml) for 48 h (Figure 1). As illustrated in Figure 1a,b, the MRBE application significantly reduced cell viability, with especially strong inhibition (>65%) at the 400 μg/ml dose (Figure 1b). As such, MRBE suppressed HepG2 cell proliferation, suggesting the potential of the extracts for treating HCC. The effects of MRBE on proliferation were examined using the MTT assay, demonstrating the strong antiproliferative action of the extract.

**FIGURE 2:**
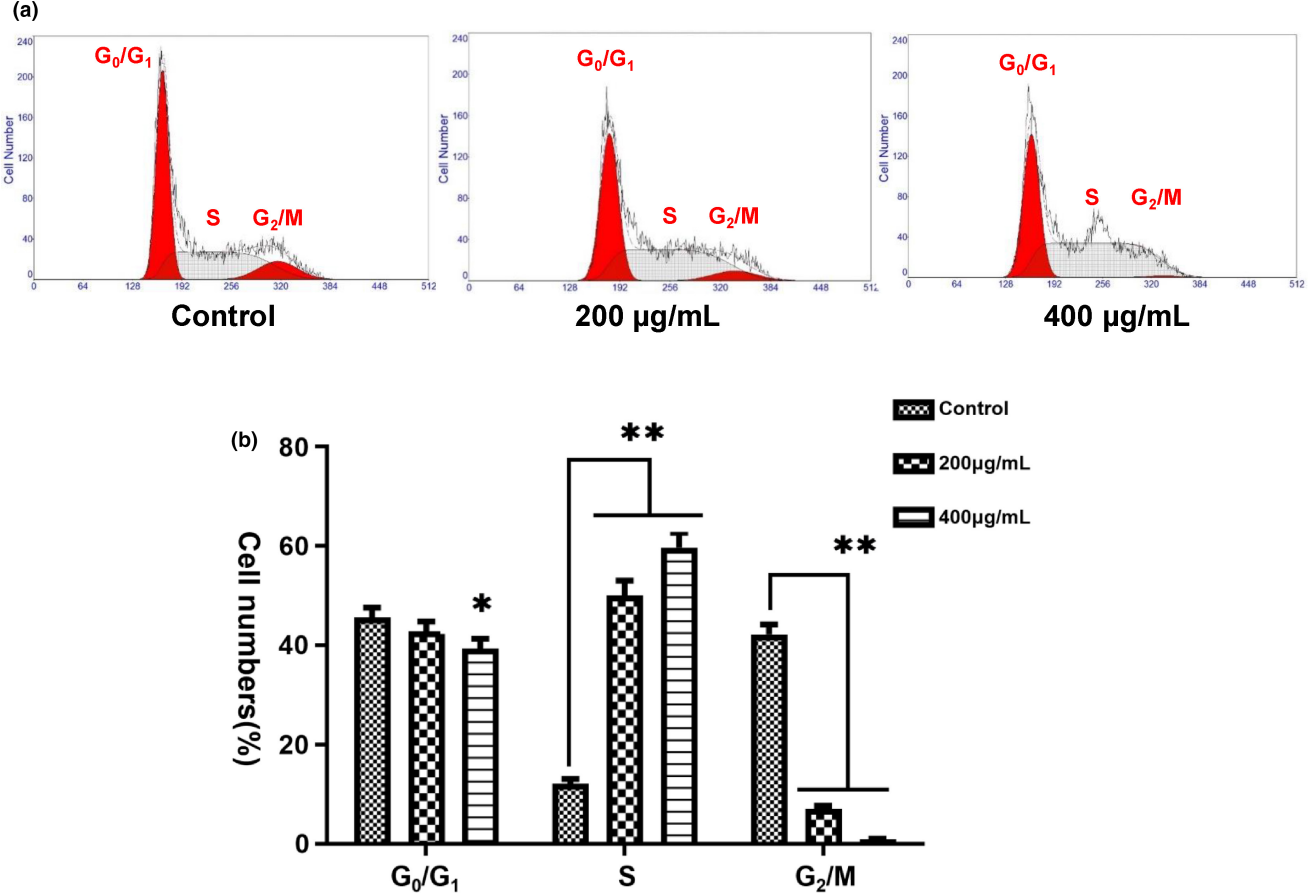
Cell cycle analysis of HepG2 cells. (a) Cell cycle distributions were based on 2N and 4N DNA content for DNA content analyses using the MultiCycle AV software. (b) Percentage of cells in different phases (G0/G1, S, and G2/M) of cell cycle. **p < .01, *p < .05 vs. control. All analyses were performed with three sample duplicates.

Abnormal proliferation is largely the result of alterations in the cell cycle and apoptotic rate. Flow cytometry further showed that the MRBE application caused S‐phase arrest, seen in increased numbers of cells in S and fewer in G0/G1 (Figure 2). This suggests that MRBE is effective in reducing proliferation in HepG2 cells.

### 3.3 MRBE induces cell S‐phase cycle arrest and apoptosis

Cell cycle is the central process underlying cellular replication, and various stages of this cycle were regulated by different genes. The mammalian cell cycle is divided into five sequential stages (G0, G1, S, G2, and M), and it normally proceeds through a series of strict regulatory checkpoints including the G1/S, S, G2/M, and mid‐ and spindle‐assembly checkpoints (Harashima et al., 2013). Only when the preceding phase is complete can the next cell cycle stage proceed. In this study, to further study the inhibitory mechanisms induced by MRBE in HepG2 cells, flow cytometry was used to investigate MRBE's effect on the cell cycle. High MRBE concentrations (200 and 400 μg/ml) led to greater numbers of cells in S phase (Figure 2). At the dose of 200 μg/ml, there were significant reductions in cells in G2/M, while cell numbers in G0/G1 declined markedly at 400 μg/ml (Figure 2). These data indicate that MRBE promotes S‐phase arrest in HepG2 cells.

**FIGURE 3:**
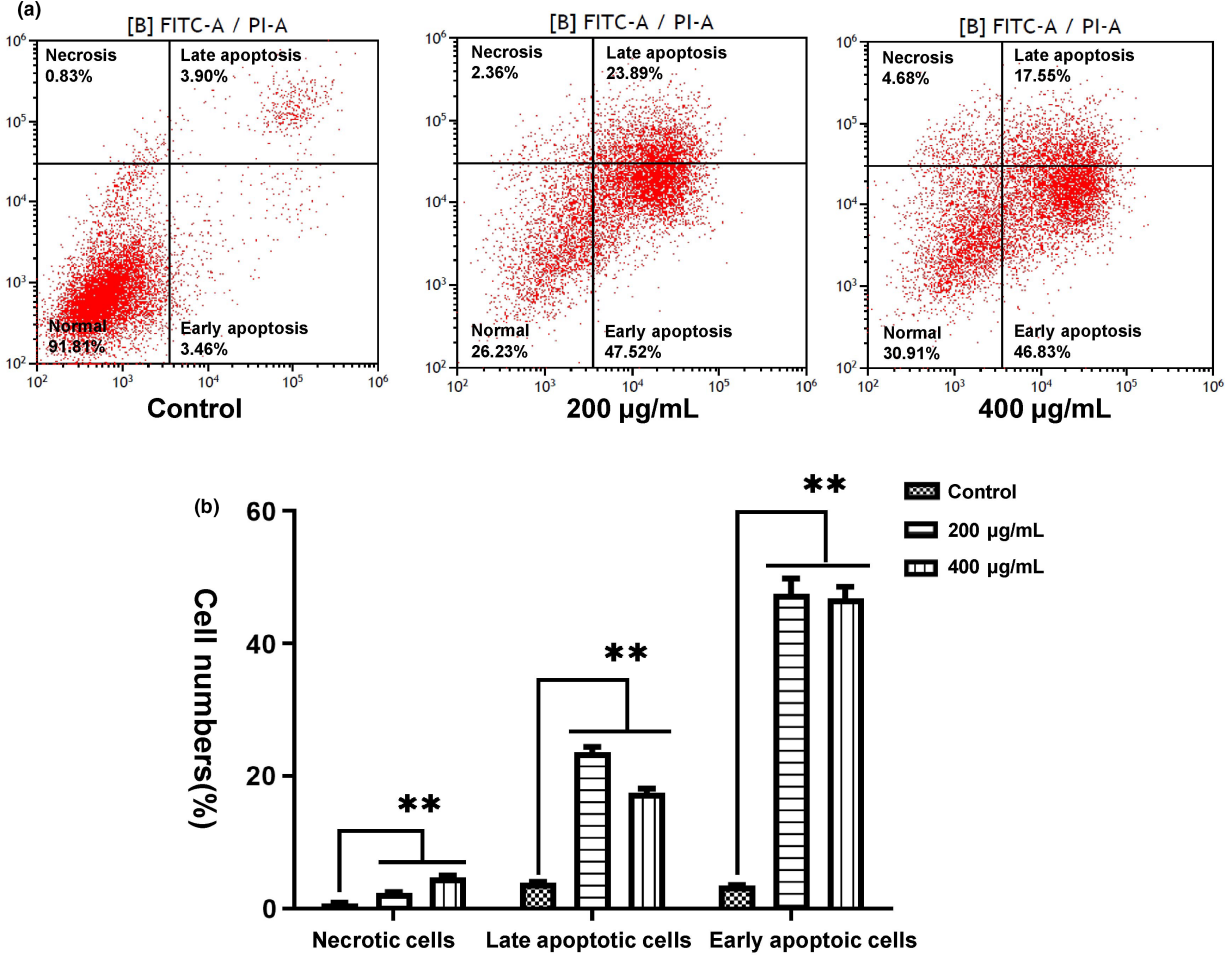
Analysis of HepG2 cell apoptosis. (a) Cells were stained with fluorescein isothiocyanate (FITC)–Annexin V and propidium iodide (PI) via flow cytometry. (b) Percentages of cells in different death types with treatments of 200 and 400 μg/ml. **p < .01 vs. control. All analyses were performed with three sample duplicates.

The effects of MRBE on apoptosis were also investigated with flow cytometry (Figure 3). MRBE treatment augmented the frequencies of both early and late apoptotic cells, as well as necrotic cells relative to controls. These findings indicate that S‐phase arrest and apoptosis may be the primary mechanism whereby MRBE suppresses the proliferation of HepG2 cells.

### 3.4 MRBE induces S‐phase arrest via p21–cyclin–CDK complex signaling pathway

**FIGURE 4:**
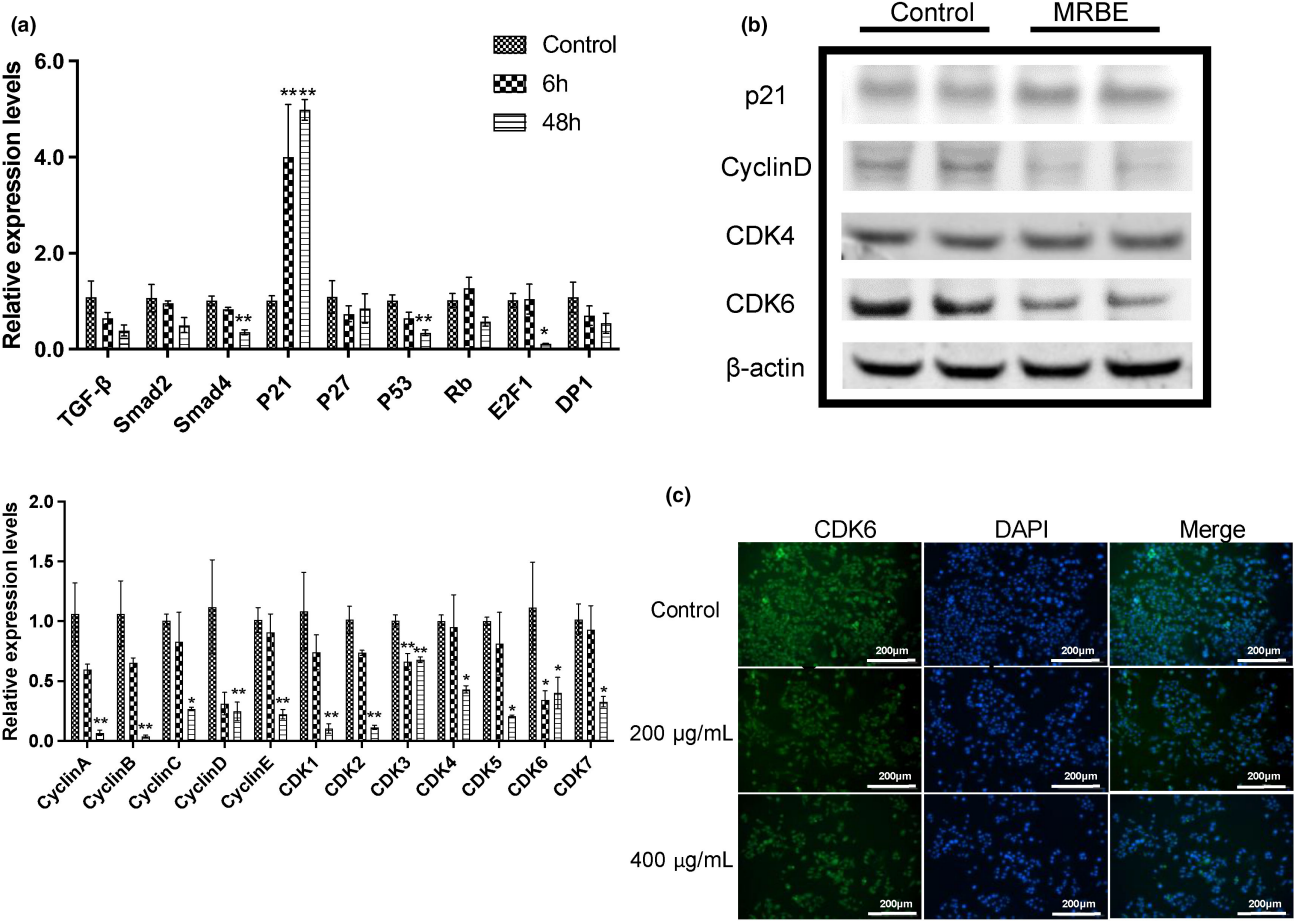
Analysis of cell cycle arrest‐associated genes in HepG2 cells. (a) qPCR analysis of cell cycle arrest‐associated genes in HepG2 cells following treatment with MRBE (400 μg/ml) for 6 h and 48 h. (b) Western blot analysis of cell cycle arrest‐associated genes in HepG2 cells following treatment with MRBE (400 μg/ml) for 48 h. (c) Immunofluorescence analysis of CDK6 staining in HepG2 cells following treatment with MRBE (40 μg/ml) for 48 h. Data are presented as the mean ± SD from four biological duplicates per group. *p < .05 and **p < .01 vs. control. MRBE, *Myrica rubra* branches extraction. *Note*: The same loading control (β‐actin) is used in both (b) and Figure 5b.

To further elucidate the antiproliferative actions of MRBE, quantitative polymerase chain reaction (qPCR) was used to investigate changes in the expression of several key cell cycle related genes. When at the highest MRBE dose (400 μg/ml), the expression level of *p21* significantly increased, whereas those of *SMAD4*, *p53*, *cyclins A/B/C/D/E*, as well as *CDK1/2/3/4/5/6/7* were downregulated (Figure 4a). No obvious changes were observed in *TGF‐β*, *SMAD2*, *p27*, *RB*, and *DP1*. Levels of the transcription factor *E2F* were also downregulated.

To confirm the correctness of gene expression by qPCR, we performed western blotting and immunofluorescence analysis. As seen in Figure 4b, the cyclin D, CDK4, and CDK6 protein levels were consistent with the results of gene expressions. Immunofluorescence showed that while there was strong staining of CDK6 in the nuclei of control cells, this was markedly reduced after MRBE treatment, together with the strength of the CDK6 green‐fluorescent signal (Figure 4c). These results suggest that MRBE may act by promoting the upregulation of p21 which, in turn, inhibits expression of the cyclin–CDK (cyclin‐dependent kinase) complex proteins.

Transforming growth factor β (TGF‐β), Smad, CDK, cyclin, E2F, and dimerization partner (DP) family of proteins control many important processes including cellular proliferation, apoptosis, differentiation, and migration (Gonzalez‐Sanchez et al., 2021). TGF‐β has been linked to HCC development after liver injury (Dituri et al., 2019; Fabregat & Caballero‐Díaz, 2018). The activation of TGF‐β signaling regulates gene encoding proteins involved in the cell cycle through Smad‐dependent transcriptional mechanisms, including the retinoblastoma (*RB*) gene and CDK inhibitors (Laiho et al., 1990; Polyak et al., 1994). CDK inhibitors include p21, p27, and p53, which belong to the CIP/Kip family and play a negative regulatory role in the cell cycle alone or in combination with one another (Orlando et al., 2015). CDK4 and CDK6 form kinase complexes with Cyclin D to phosphorylate retinoblastoma (Rb) family proteins, promoting cellular progression from G0 to G1, driving the release of E2F, Cyclin A, and Cyclin E from Rb protein inhibition, and thereby promoting transcription. CDK2 facilitates cell progression from G1 to S by complexing with cyclins A and E, while *CDK1* forms kinase complexes with cyclins A and B, resulting in progression from S to M phase (Sherr & Roberts, 1999). Taken together, this analysis showed upregulation of p21 and downregulation of the Cyclin D–CDK4/6, Cyclin A/E–CDK2, and Cyclin A/B–CDK1 complexes, while E2F levels were diminished possibly due to the reductions in Cyclin A/B and CDK1. Therefore, these findings indicate that MRBE treatment upregulates *p21* expression to inhibit cyclin–CDK complexes, thereby inducing the S‐phase arrest.

### 3.5 MRBE induces apoptosis by downregulating Bcl‐2 and ERK1 expression

**FIGURE 5:**
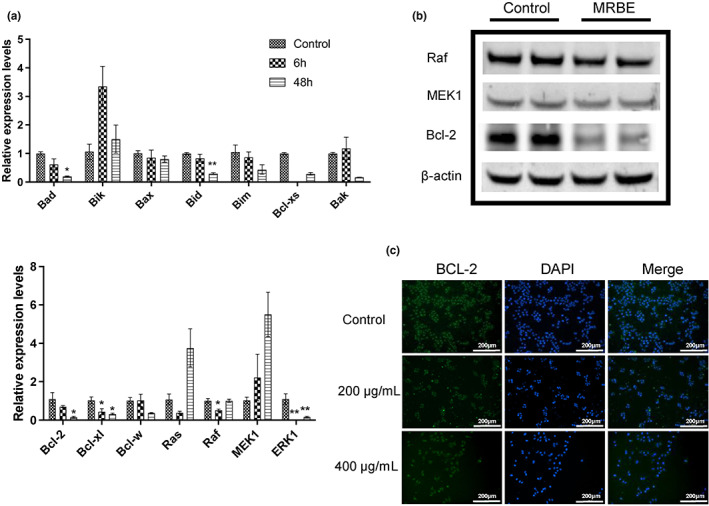
Analysis of apoptosis‐associated genes in HepG2 cells. (a) qPCR analysis of apoptosis‐associated genes in HepG2 cells following treatment with MRBE (400 μg/ml) for 6 h and 48 h. (b) Western blot analysis of apoptosis‐associated genes in HepG2 cells following treatment with MRBE (400 μg/ml) for 48 h. (c) Immunofluorescence analysis of BCL‐2 in HepG2 cells following treatment with MRBE (400 μg/ml) for 48 h. Data are presented as the mean ± SD from four biological duplicates per group. *p < .05 and **p < .01 vs. control. MRBE, *Myrica rubra* branches extraction. *Note*: The same loading control (β‐actin) is used in both Figure 4b and (b).

Next, qPCR was used to examine the levels of several apoptotic genes. The relative mRNA levels of pro‐apoptotic genes (*BAD*, *BID*, *BCL‐2*, *BCL‐XL*, *RAF*, and *ERK1*) were attenuated after MRBE application. Specifically, the expression of *RAF* was significantly decreased at 6 h, while levels of *BAD*, *BID*, and *BCL‐2* were markedly reduced after 48 h, and *BCL‐XL* and *ERK1* were attenuated at both 6 and 48 h (Figure 5a). However, no differences were observed in *BIK*, *BAX*, *BIM*, *BAK*, *BCL‐XS*, *BCL‐W*, *RAS*, and *MEK1* (Figure 5a). Levels of the anti‐apoptotic genes *BCL‐2* and *ERK1* were markedly reduced after MRBE treatment for 48 h (Figure 5a). Western blotting confirmed that these alterations were in accordance with the protein levels (Figure 5b). Moreover, immunofluorescent staining for Bcl‐2 showed a marked reduction in cell density after application of 400 μg/ml MRBE for 48 h, with a similar decline in the green‐fluorescent Bcl‐2 signal (Figure 5c). Together, these findings indicated that MRBE treatment may lead to apoptosis by downregulating the expression of *BCL‐2* and *ERK1* in HepG2 cells.

Apoptosis refers to the programmed death of cells via a regulated pathway that maintains the stability of the internal environment, and many cancer cells fail to normally undergo apoptotic death, leading to uncontrolled proliferation (Wu et al., 2008). The process is largely controlled by Bcl‐2 proteins, which are separated into three primary groups. Group 1 includes the anti‐apoptotic Bcl‐w, Bcl‐xl, and Bcl‐2 proteins, while Group 2 includes the pro‐apoptotic Bax and Bak proteins, and Group 3 includes the BH3 proteins Bik, Bid, Bim, and Bad (Adams et al., 2019; García‐Sáez, 2012; Schafer et al., 2009). The equilibrium between anti‐ and pro‐apoptotic proteins is strictly controlled, affecting cell survival (Klanova & Klener, 2020). In addition, aberrant activation of the Ras/Raf/MEK/ERK signaling pathway is involved in the proliferation, differentiation, survival, and apoptosis of HCC cells. In the present study, MRBE induced HepG2 cell apoptosis through a mechanism correlated with significant decreases in anti‐apoptotic Bcl‐2 and Bcl‐xl expression. Such decreases have the potential to induce apoptosis even in the context of the slight downregulation of some pro‐apoptotic proteins. In addition, the levels of *Raf* and *ERK*, also known to promote apoptosis, were markedly reduced in these cells. While these results are preliminary, they highlight promising directions for further research of MRBE.

## 4 CONCLUSIONS

This investigation demonstrated that alcohol extracts of Chinese bayberry branch were able to effectively inhibit HepG2 cell proliferation through S‐phase arrest. This action appears to be accomplished by the activation of p21–‐Cyclin–CDK complex signaling pathway. MRBE also promoted apoptosis by attenuating expression of Bcl‐2 and the proteins of the Raf/ERK1 (extracellular signal‐regulated kinase 1) signaling pathway. This suggests that MRBE may be a useful candidate for drug development efforts aimed at preventing and treating HCC. This study provided the potential roles of waste branches of Chinese bayberry, and it could not only realize the rational utilization of resources, turn waste into treasure, and reduce pollution, but also expand the bayberry industry chain.

## CONFLICT OF INTEREST

All authors declare no conflict of interest.

## ETHICAL APPROVAL

This study does not involve any human or animal testing.

## Supporting information


**Table S1.** Primers used in the study.

## Data Availability

The data will be available on request.
