# Supplementary material for: Alcohol extracts of Chinese bayberry branch induce S‐phase arrest and apoptosis in HepG2 cells
Source: Food Sci Nutr. 2022 Oct 2;11(1):493–503. doi: 10.1002/fsn3.3080 (PMC9834848; doi:10.1002/fsn3.3080)
Supplement: Supplementary file 1 — Table S1. Primers used in the study. [file FSN3-11-493-s001.docx]

| **Gene** | **Forward primer (5'–3')** | **Reverse primers (5'–3')** |
| --- | --- | --- |
| *TGFB* | GGACCAGTGGGGAACACTAC | AGAGTCCCTGCATCTCAGAGT |
| *Smad2* | CCATACCAAGGTCTCTTGATGGT | ACTGTGAAGATCAGGCCAGC |
| *Smad4* | GCAGTCCTACTTCCAGTCCAG | CCCATCTGAGTCTAATGCTACC |
| *p21* | AGCGACCTTCCTCATCCACC | AAGACAACTACTCCCAGCCCCATA |
| *p27* | AAAAGCAACAGAAACCTATCCTCAC | ATTCAAAACTCCCAAGCACCTC |
| *p53* | AGCTTTGAGGTGCGTGTTTGTG | TCTCCATCCAGTGGTTTCTTCTTTG |
| *Rb* | TTGTAACGGGAGTCGGGAGA | CAGCGAGCTGTGGAGGAG |
| *E2F1* | CACTTTCGGCCCTTTTGCTC | GTGCTCTCACCGTCCTACAC |
| *DP1* | ATGGCTCAGGGAACTGTTGG | GGTCACTGGCAGAGAACCTT |
| *Cyclin A* | TGGAAAGCAAACAGTAAACAGCC | GGGCATCTTCACGCTCTATTT |
| *Cyclin B* | GCAGCACCTGGCTAAGAATGT | GCCTTGGCTAAATCTTGAACT |
| *Cyclin C* | GCAGGACATGGGCCAAGAAGA | TATCCACAGAAAGCTCAGCAAACCA |
| *Cyclin D* | CCCTCGGTGTCCTACTTCAAATGT | GGAAGCGGTCCAGGTAGTTCAT |
| *Cyclin E* | GCCAGCCTTGGGACAATAATG | CTTGCACGTTGAGTTTGGGT |
| *CDK1* | GGATGTGCTTATGCAGGATTCC | CATGTACTGACCAGGAGGGATAG |
| *CDK2* | CCAGGAGTTACTTCTATGCCTGA | TTCATCCAGGGGAGGTACAAC |
| *CDK3* | CCAGCTCTTTCGTATCTTTCGT | TTCCTGGTCCACTTAGGGAAG |
| *CDK4* | ATGGCTACCTCTCGATATGAGC | CATTGGGGACTCTCACACTCT |
| *CDK5* | GGAAGGCACCTACGGAACTG | GGCACACCCTCATCATCGT |
| *CDK6* | TCTTCATTCACACCGAGTAGTGC | TGAGGTTAGAGCCATCTGGAAA |
| *CDK7* | ATGGCTCTGGACGTGAAGTCT | GCGACAATTTGGTTGGTGTTC |
| *Bad* | TTGGGGTGAGACCTGTGCG | CTCAGTCTCCCCTCAGAACCC |
| *Bik* | CATTCCAAAGAATCGAAGTCC | AATGTCTGAAGTAAGACCCCTCT |
| *Bax* | CCCGAGAGGTCTTTTTCCGAG | CCAGCCCATGATGGTTCTGAT |
| *Bid* | AGGAAGCCAAACACCAGTAGG | TGTGAACCAGGAGTGAGTCGG |
| *Bim* | TTTCTTGCGTTTCTCAGTCCG | CGCCACTACCACCACTTGATT |
| *Bcl-xs* | TCCCCATGGCAGCAGTAAAG | TCCACAAAAGTATCCTGTTCAAAGC |
| *Bak* | GCAGGCTGATCCCGTCC | CTGCGGAAAACCTCCTCTGT |
| *Bcl-2* | GGTGGGGTCATGTGTGTGG | CGGTTCAGGTACTCAGTCATCC |
| *Bcl-xl* | GCTGGGACACTTTTGTGGAT | GAGCCCAGCAGAACTACACC |
| *Bcl-w* | CTTTGTCTTTGGGGCTGCAC | CCATCCACTCCTGCACTTGT |
| *Ras* | AAGTGTGTGCTCTCCTGACG | CACAAGGGAGGCTGCTGAC |
| *Raf* | CAACTGATTGCACTGACTGCC | CCTGGAATTGCTCTGGGGTT |
| *MEK1* | CTTCGCAGAGCGGCTAGG | CAGCCCGGGATTCCCTTC |
| *ERK1* | GAGATGGATGTGGGTTCCAGTC | TTCTCATCGTAGCCACTCACC |
| *GAPDH* | GGACCTGACCTGCCGTCTAG | GTAGCCCAGGATGCCCTTGA |

Table S1 Primers used in the study
